# Progesterone's Effects on Cognitive Performance of Male Mice Are Independent of Progestin Receptors but Relate to Increases in GABA_A_ Activity in the Hippocampus and Cortex

**DOI:** 10.3389/fendo.2020.552805

**Published:** 2021-01-11

**Authors:** Cheryl A. Frye, Vincent F. Lembo, Alicia A. Walf

**Affiliations:** ^1^Department of Psychology, The University at Albany-SUNY, Life Sciences, Albany, NY, United States; ^2^Department of Biological Sciences, The University at Albany-SUNY, Life Sciences, Albany, NY, United States; ^3^The Center for Neuroscience Research, The University at Albany-SUNY, Life Sciences, Albany, NY, United States; ^4^The Center for Life Sciences Research, The University at Albany-SUNY, Life Sciences, Albany, NY, United States; ^5^Institute of Arctic Biology, University of Alaska–Fairbanks, Fairbanks, AK, United States; ^6^Department of Chemistry, University of Alaska–Fairbanks, Fairbanks, AK, United States; ^7^IDeA Network of Biomedical Excellence (INBRE), University of Alaska–Fairbanks, Fairbanks, AK, United States; ^8^Comprehensive Neuropsychological Services, Albany, NY, United States; ^9^Department of Cognitive Science, Rensselaer Polytechnic Institute, Troy, NY, United States

**Keywords:** brain-derived neurotrophic factor, prefrontal cortex, allopregnanolone, hippocampus, object recognition, T-maze, memory

## Abstract

Progestogens' (e.g., progesterone and its neuroactive metabolite, allopregnanolone), cognitive effects and mechanisms among males are not well-understood. We hypothesized if progestogen's effects on cognitive performance are through its metabolite allopregnanolone, and not actions via binding to traditional progestin receptors (PRs), then progesterone administration would enhance performance in tasks mediated by the hippocampus and cortex, coincident with increasing allopregnanolone concentrations, brain derived neurotrophic factor (BDNF) and/or muscimol binding of PR knock out (PRKO) and wild-type PR replete mice. **Experiment 1**: Progesterone (4 mg/kg, subcutaneously (SC; *n* = 12/grp), or oil vehicle control, was administered to gonadally-intact adult male mice PRKO mice and their wild-type counterparts and cognitive behaviors in object recognition, T-maze and water maze was examined. Progesterone, compared to vehicle, when administered post-training increased time investigating novel objects by the PRKO and wild-type mice in the object recognition task. In the T-maze task, progesterone administration to wild-type and PRKO mice had significantly greater number of spontaneous alternations compared to their vehicle-administered counterparts. In the water maze task, PRKO mice administered vehicle spent significantly fewer seconds in the quadrant associated with the escape platform on testing compared to all other groups. **Experiment 2**: Progesterone administered to wild-type and PRKO mice increased plasma progesterone and allopregnanolone levels (*n* = 5/group). PRKO mice had higher allopregnanolone levels in plasma and hippocampus, but not cortex, when administered progesterone and compared to wild-type mice. **Experiment 3**: Assessment of PR binding revealed progesterone administered wild-type mice had significantly greater levels of PRs in the hippocampus and cortex, compared to all other groups (*n* = 5/group). Wild-type mice administered progesterone, but not vehicle, had increased BDNF levels in the hippocampus, but not the cortex, compared to PRKOs. Wild-type as well as PRKO mice administered progesterone experienced significant increases in maximal GABA_A_ agonist, muscimol, binding in hippocampus and cortex, compared to their vehicle-administered counterparts. Thus, adult male mice can be responsive to progesterone for cognitive performance, and such effects may be independent of PRs trophic actions of BDNF levels in the hippocampus and/or increases in GABA_A_ activity in the hippocampus and cortex.

## Introduction

Our understanding of progesterone, a gonadal hormone that is produced primarily by the ovaries in females, as well as progestin receptors (PRs) functioning, has primarily come from studies in females (1–3). Although progesterone has always been considered a “female-typical hormone,” adult male rodents produce progesterone in the testes and adrenal cortex (4, 5). Male rodents have circulating levels of progesterone around 1.5–2 ng/mL (6, 7), compared to a range of 3–35 ng/mL in females that is seen throughout the estrous cycle (8). Males, compared to females, have higher levels of steroid receptor co-activators, which enhance steroid hormone action in many brain regions (9). Of note, across species, both males and females have early exposure to maternal progesterone, by which brain functioning is organized. “Male-typical” hormones, such as androgens are derived from a cholesterol-based pro-hormone, progesterone. Thus, despite conceptualization of progesterone as a female hormone, the extent to which adult males respond to progesterone is an important question.

Progesterone can exert beneficial effects for cognitive performance; however, most of the work on progesterone's cognitive effects has involved female subjects. Compared to other treatments, progesterone to rodents assessed in the Morris water maze reduced latencies to the hidden platform, increased platform crossings, and time spent swimming in the quadrant area where the platform had been during training (10). Progesterone improved reference memory acquisition and reversal learning in the Morris water maze task, compared with vehicle treatment (11). In addition, young and aged rodents administered progesterone, or its neuroactive metabolite, allopregnanolone, performed significantly better in the object recognition, object placement, T-maze, and water maze tasks compared to other groups (12, 13). Progesterone can have memory-enhancing effects among young adult mice in condition place preference, inhibitory avoidance and other tasks that may be mediated by several brain regions, including the hippocampus, prefrontal cortex (PFC), amygdala, nucleus accumbens, and cerebellum (14, 15). Thus, progesterone has beneficial effects to improve cognitive performance of female rodents across a variety of tasks. The question remains about the responsiveness of males to progesterone on cognitive performance.

To understand the role and brain targets of progestogens for cognitive performance among males, different mechanisms of actions of progesterone and its metabolic allopregnanolone should be considered. Unlike allopregnanolone, progesterone binds with high affinity to intracellular PRs (16). Progestin receptors have been localized to brain targets for learning/memory effects of progesterone in the hippocampus (17) and the frontal cortex (18). However, progesterone may be exerting its effects through its metabolite, allopregnanolone, which has greater affinity for γ-aminobutyric acid (GABA_A_) receptors. Allopregnanolone alters functioning of many neurotransmitter targets, rather than binding to PRs, when in physiological concentrations (19–21). Female mice, administered allopregnanolone or those that were administered progesterone and could metabolize this to allopregnanolone, performed significantly better in the object recognition, object placement, T-maze and water maze tasks compared to female mice administered vehicle (12). In addition, rodents administered allopregnanolone, perform better in the water maze, a delayed nonmatching-to-sample Y-maze task, object recognition and object placement tasks, and conditioned aversion tasks and have enhanced conditioned place preference compared to controls (22–27). Moreover, PR knockout (PRKO) mice, which lack PRs throughout development (28), have been used. For example, young and/or aged PRKO and wild-type mice have increased sexual responding, decreased anxiety-like behavior, and enhanced cognition following progesterone administration, despite PRKO mice having low levels of cortical PR binding (29, 30). Cognitive enhancement among both PRKO and wild-type mice administered progesterone suggests that PRs are not necessary for progesterone's beneficial effects on cognitive performance. Thus, progesterone's beneficial effects across various cognitive tasks may be related to the capacity to produce allopregnanolone, rather than actions at PRs among females. Of interest is the effects among males.

Another non-PR target to be considered is brain-derived neurotrophic factor (BDNF). BDNF is produced both in neurons and glial cells (31, 32). BDNF is of interest as a marker of neural plasticity, which may play a role in synaptic plasticity and learning/memory (33, 34). There is strong evidence to support the role of BDNF in synaptic plasticity and cognitive function (35) and as such, alterations in its function and/or expression have been implicated in the pathophysiology of aged-related neurodegenerative diseases including Alzheimer's disease, Parkinson's disease, seizure disorder, major depression (34, 36–45), and a variety of stressors/events [e.g., ischemia, hypoglycemia, stressor exposure, etc. (46, 47)]. Restoring BDNF expression and/or function may be therapeutic. Furthermore, there is evidence that progesterone and other hormones have enhancing effects on BDNF expression. BDNF levels are increased and are associated with administration of progesterone and/or cognitive enhancement (12, 33, 34, 48–53). Thus, BDNF may play a role for cognitive enhancement following progesterone administration.

An important question is the responsiveness of progesterone in a mouse model that should be less sensitive to progesterone effects (adult males, low levels of progesterone, and no PRs). Notably, young males in some cases do not respond to progesterone as females do. For example, duration spent immobile in the forced swim task is not reduced with progesterone administration in young males to the same extent that it is in age-matched females; this sex difference is no longer apparent in aged mice (54). We hypothesized if progesterone's effects on learning/memory are through its metabolite allopregnanolone, and not due to traditional actions via binding to PRs, progesterone administration post-training will enhance performance in tasks mediated by the hippocampus and cortex, coincident with increasing allopregnanolone concentrations in the hippocampus and cortex, and increase BDNF levels or activity of GABA_A_ receptors of both PRKO and wildtype mice. To test this, gonadally-intact male wild-type and PRKO mice were administered progesterone and/or oil vehicle and exp 1: cognitive behaviors (object recognition, T-maze and Water maze), exp 2: neuroendocrine factors (plasma, hippocampal and cortical progesterone and allopregnanolone levels), and trophic factors, PR binding, BDNF levels in the hippocampus and cortex, and GABA_A_ activity in the hippocampus and cortex were assessed.

## Methods and Materials

The methods utilized for animal husbandry, determination of WT vs. PRKOs, drug administration, behavioral testing, euthanasia and tissue collection in the murine subjects in this study were approved by the Institutional Animal Care and Use Committee at the University at Albany.

### Animal Housing

Subjects were adult (8–10 weeks old), male mice. Mice were group-housed (4–5 per cage) in polycarbonate cages (26 × 16 × 12 cm) in a temperature-controlled room (21 ± 1°C) in the core Laboratory Animal Care Facility at the University at Albany. The housing room was on a 12/12-h reversed light cycle (lights off 8:00 a.m.-8:00 p.m.). Mice had continuous access to Purina Mouse Chow and tap water in their home cages and were assessed during their dark phase. There were 50/12-13 mice group in one cohort and 20/5 mice group in another cohort. The first cohort of 5 per group was done to examine physiological measure around 15 generations of back crossings to bring the PRKO mice from their 129 background strain onto the c57UA strain, which were c57 mice that had been subjected to random and frequent fire alarms with changes in air pressure for 4 years.

### Mouse Strain and Genotyping

PRKO mice that were back crossed onto a c57 background are not distinguishable based upon any obvious phenotypic or behavioral characteristics from c57 controls. As such, another member of the laboratory conducted genotyping, as described below, and randomly assigned them to groups, which were unknown to the individuals that were testing the animals. Subjects were wild-type (+/+) or (-/-) PRKO mice, congenic on C57BL/6 background, that were derived from heterozygous (+/–) breeder pairs from a colony that was maintained at the University at Albany. These mice were developed by Bert O'Malley's laboratory [Baylor College of Medicine, Houston, Texas; (29, 55)]. Typical polymerase chain reaction (PCR) methods, modified from Jackson Laboratory's published protocol, were utilized to determine the genotype of mice (54, 56). Briefly, genomic DNA was isolated from tail snips and analyzed by PCR. PCR was performed by denaturing the DNA at 95°C for 5 min, followed by 30 cycles of amplification: 94°C for 1 min, 60°C for 1 min, 72°C for 1 min, and a final primer extension step at 72°C for 10 min. The following PR specific primers were used: P1 (5′TAGACAGTGTCTTAGACTCGTTGTTG-3′), P2 (5′GATGGGCACATGGATGAAATC-3′), and a neo gene specific primer, N2 (5′GCATGCTCCAGACTGCCTTGGGAAA-3′). Primers were obtained from Integrated DNA Technologies (Coralville, IA). Bands of ~565 and 500 base pairs were amplified for wild-type and PRKO mice, respectively. PRKO and wild-type mice were randomly assigned to receive progesterone or vehicle as described below. The individual who was testing the animals was blind to the genotype of all animals and the vehicle or progesterone administration condition.

### Progesterone Administration

Crystalline progesterone was obtained from Steraloids, Newport, RI and dissolved in vegetable oil vehicle. Intact male mice were randomly assigned to receive progesterone (4 mg/kg) or vehicle (vegetable oil) by subcutaneous injection (SC) in the nape of the neck 1 h before behavioral testing in the T-maze, immediately after training in the single trial of the object recognition task, and after the last training trial in the water maze (57).

### Behavioral Testing

Wild-type and PRKO mice were assigned to one hormone condition (vehicle or progesterone) and then, tested once per week in each of the behavioral tasks described below. Behavioral data were collected simultaneously by an experimenter (T-maze), the Any-Maze tracking system (Stoelting, Wood Dale, IL; object recognition), and/or both methods (water maze). On the day when mice were trained and tested, they were transported in their home-cages on a cart to the testing area. Mice were singly-housed in a clean cage immediately before training and until the last testing trial, when they were returned to their home-cage in the vivarium.

#### Object Recognition

In the object recognition task, mice were trained with two identical objects, i.e., a plastic toy block or a bottle, that were placed in an open field. The objects used were those that mice showed a high and similar degree of investigating during a single, 3 min training trial (58). There were no significant differences between genotypes or treatment group for time spent investigating objects during training in the object recognition task [left object: WT: 12.1 ± 2.2 s (SEM), PRKO: 10.2 ±1.4 s (SEM); right object: WT: 11.1 ± 1.9 s (SEM), PRKO: 11.9 ±2.3 s (SEM); training data are from mice that were trained before receiving treatment]. The durations spent within 5 cm of the object, directly in contact, investigating and/or orienting toward the objects were automatically recorded using Any-Maze for the training and testing trials. Immediately after training, mice were injected with vehicle and/or progesterone. Mice were tested in this task 4 h after training. During testing, there was a novel object and a familiar object (i.e., the object that mice had been trained with) and mice freely explored in the testing chamber for 3 min. The duration of time the mice spent exploring the familiar and novel objects were recorded.

#### T-maze

Spontaneous alternation was assessed in the T-maze, which has a clear Plexiglas start box connected to a start arm (30.5 × 9 × 7 cm) and two goal arms (17.8 × 9 × 7 cm). Mice were placed in the start box 1 h after vehicle or progesterone treatment. The door was opened and following one forced trial (where either the left or right side was blocked in a random fashion), the number of spontaneous alternations made to each goal arm was assessed for 13 consecutive trials (max latency = 900 s). Each of the 13 trials consisted of the mouse fully returning to the start arm and then, entering the right or left goal arm (13, 59). Data were hand-collected by an experimenter and videos of trials were recorded using Any-Maze or a video-camera. The index of performance in this task is the number of successful alternating trials out of 13 possible trials.

#### Water Maze

The water maze was filled with 25°C tap water and was made opaque by the addition of white non-toxic tempera paint. Mice were habituated to the maze by allowing them to swim in the water maze with the hidden platform (8 × 8 cm) in it. After 1 min, mice were placed on the hidden platform for 10 s. Following habituation, mice were trained in twelve 1-min trials which were organized into 3 blocks of 4 trials with a randomized starting position in the maze represented during each of these 4 trials in the block. There were 3 different starting positions in the maze. In each trial, mice had 60 s to find the clear platform in the opaque water (hidden platform). Latencies to find the platform and distance traveled were recorded simultaneously by the experimenter and the Any-Maze tracking program. Each block of trials had a 30 min inter-trial interval. Mice were injected with vehicle or progesterone immediately after the last training trial. Before the probe trial to assess spatial memory, the hidden platform was removed from the water maze. Thirty minutes following vehicle or progesterone administration, mice were returned to the water maze at a random position. The latency to return to the quadrant that had the platform, and the duration of time spent in that quadrant, were the indices of cognitive performance in this task. Immediately after the probe trial, mice were tested in a cued trial of the water maze to assess their ability to swim to a platform in the maze. During this trial, the latency of mice to swim to a platform that is made visible and cued is determined to rule out the ability to perform the task (13, 30). There were no differences between groups in these measures (data not shown).

### Tissue Collection

Immediately after testing in the water maze, mice were euthanized by cervical subluxation and decapitation. Whole brains were collected from mice and stored frozen at −70°C until brain regions were processed for enzyme-linked immunosorbent assays (ELISA), progesterone, allopregnanolone, BDNF, PR binding and muscimol binding. The cortex and hippocampus were grossly dissected from the whole brain on ice prior to steroid and BDNF measurement.

### Sample Preparation

The cortex and hippocampus were dissected out and homogenized with a pestle in 500 microliters of distilled water in a microcentrifuge tube and centrifuged for 10 min at 3,000 x g. Protein concentrations in each sample were measured using a Nanodrop Spectrometer (Thermo Scientific, Federal Way, WA).

### Allopregnanolone, Progesterone and BDNF ELISAs

Analyses of allopregnanolone and progesterone were per standard methods of the ELISA kits purchased from Arbor Assays (Ann Arbor, MI). Fifty microliters of homogenized sample were added to each well.

Analyses of BDNF were per standard methods of the Emax Immunoassay system [Promega, Fisher Scientifics; (12, 60, 61)]. Brain homogenates were homogenized in 10 microliters of cell lysis buffer (Qiagen) with a pestle in a microcentifuge tube. Fifty microliters of these prepared homogenates were diluted in 4 volumes of Dulbecco's Phosphate-Buffered Saline (Fisher Scientific). Diluted samples were acid-treated by adding 1 microliter of 1 N HCl, incubating for 15 min at room temperature, and then neutralizing the samples by adding 1 microliter of 1 N NaOH.

For allopregnanolone, progesterone and BDNF ELISA, 50 microliters of prepared Detection Reagent A was immediately pipetted into wells. Plates were shaken, mixed and incubated for 1 h at 37°C. Samples were then aspirated and washed 3 times with 350 microliters of 1x wash buffer. Any remaining liquid from all wells was removed completely by snapping the plate onto absorbent paper. Next, 100 microliters of prepared Detection Reagent B was pipetted into each well and incubated for 30 min at 37°C. Plate was then aspirated and washed 5 times with 350 microliters of 1x wash buffer and any remaining liquid was removed from wells by snapping the plate onto absorbent paper. Then, 90 microliters of substrate solution was pipetted into each well and incubated for 15–25 min at 37°C and placed in the dark where the liquid turned blue. Lastly, 50 microliters of stop solution was pipetted into each well and gently tapped. The addition of the stop solution turned the liquid yellow. Immediately after, the plate was read at 450 nm on a microplate reader (Bio-tek, Thermo Scientific, Federal Way, WA).

### Progestin Receptor Binding

Progestin receptors in hippocampus and cortex were investigated in *n* = 5 mice per group to confirm that backcrossing PRKOs to make them congenic on our c57UA strain did not alter their brain levels of progestin receptors. We used the tritiated synthetic competitive binding assay as previously described (54, 55) with RU5020 (promegestrone; 17α, 21-dimethyl-19-nor-pregna-4,9-diene-3,20-dione), which has a Kd of 0.4 nM for progestin receptors (62). Progestin receptors are found in pituitary, reproductive tract and most estrogen receptor-containing brain regions (62). There are also progestin receptor sites in brain areas lacking estrogen receptors, such as the cerebral cortex, which are similar to those induced by estradiol.

### Muscimol Binding

As per our previous methods (63), tissues were thawed and resuspended in 0.05 M Tris-HC1 buffer to a protein concentration of 0.8 mg protein per tube in a final volume of 0.5 ml. [^3^H] muscimol (NET 574, spec. act. 14.72 Ci/mmol, New England Nuclear, Boston, MA, USA) at 10–100 nM concentrations was added and incubation was continued at 4°C for 30 min. Non-specific binding was determined by addition of 1 mM cold GABA as a displacer of bound [3H] muscimol. The bound and free fractions were separated by vacuum filtration through GF/C glass filters and washed twice very quickly with 0.05 M Tris buffer [89]. Filters were placed in scintillation vials to dry overnight. The next day, scintillation cocktail was added and the vials were counted for radioactivity and degradations per minute were calculated. The E_max_ of specific muscimol binding was used as the variable of interest.

### Statistical Analyses

For all measures except the T-maze task, two-way ANOVAs were used to determine effects of hormone condition (vehicle or progesterone) and genotype (wild-type or PRKO). Two subjects were removed from the main cohort, as they varied more than 2 standard deviations from the mean in their group. One subject was from the wild-type progesterone group and the other was from the wild-type prko group. This resulted in an equal number of observations across groups (*n* = 12/group) and no question as to the adherence of the assumptions of the premises of the ANOVAs. A *X*^2^-square test of independence with Yates correction was performed to examine the relationship between progesterone and genotype and the ability to alternate in the T maze. The α level for statistical significance was *p* ≤ 0.05. Fisher's *post-hoc* tests were used to examine group differences.

## Results

### Progesterone to PRKO and Wild-Type Male Mice Enhances Object Recognition

**Figure 1:**
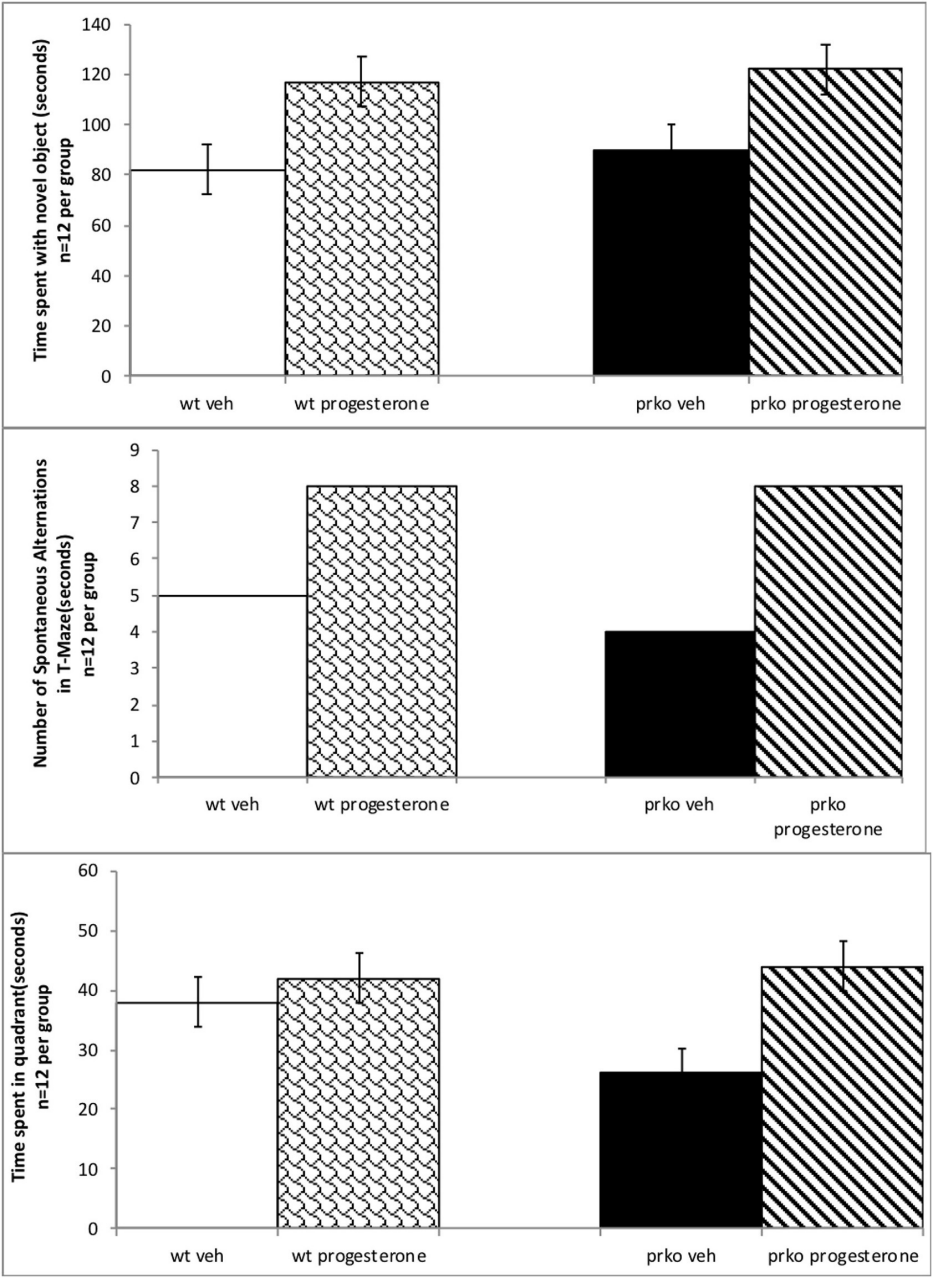
Results from the vehicle wildtype mice are represented by white bars, P_4_ wildtype by stippled bars, vehicle PRKO by black bars, and P_4_ PRKO mice are represented by diagonally striped bars. There are 12 animals per experimental group. The **top** panel represents mean time (in secs) spent with novel object (±S.E.M.). Wildtype or PRKO male mice administered P_4_ had enhanced cognitive performance in the object recognition task compared to vehicle administration among wild-type and PRKO mice. The **middle** panel indicates the mean number of spontaneous alternations (±S.E.M.) in the T-maze out of 13 trials. Wildtype or PRKO male mice administered P_4_ had ac greater number of spontaneous alterations in the T-maze compared to vehicle administered groups. The **bottom** panel represents the mean number of seconds spent in the quadrant (±S.E.M.) where the hidden platform had been in previous trials. PRKO vehicle mice spent significantly less time in the quadrant than did all other groups.

Progesterone administered mice spent significantly more time with the novel object during testing in the object recognition task. Wild-type and PRKO mice administered progesterone spent more time spent with the novel object compared to their vehicle administered counterparts [(F_(1, 44)_ =22.88, *P* < 0.001) see Figure 1, top]. There were neither effects of genotype, nor significant interactions of genotype and progesterone, for time spent with the novel object during testing.

### Progesterone to PRKO and Wild-Type Male Mice Results in More Spontaneous Alternation in the T-maze Task

A chi-square test of independence was performed to examine the relationship between progesterone and genotype and the ability to alternate in the T maze. The relationship between these variables was significant, according to *X*^2^ with Yates correction (4, *N* = 48) = 8.9, *p* = 0.02. Mice, irrespective of wild type or PRKO, administered progesterone made significantly greater number of alternations in the T-maze than did vehicle-administered mice. Indeed, progesterone administered mice made approximately twice the number of spontaneous alternations than did their vehicle-administered counterparts. See Figure 1, middle.

### Progesterone to PRKO and Wild-Type Male Mice in the Water Maze Task

Hormone condition and genotype interacted, such that PRKO, vehicle-administered mice had longer latencies to find the quadrant where the platform had been, compared to all other groups [F_(3, 44)_ = 22.87, *P* < 0.0001]. Progesterone and genotype also interacted in that PRKO, vehicle-administered mice, spent less time in the quandrant where the platform had been located compared to all other groups [F_(3, 44)_ = 13.56, *P* < 0.0001] See Figure 1, bottom. There were no significant differences for the time spent in the quadrant of the hidden platform during the probe trial and the latency to find the platform in the cued trial indicating initial performance variables were not a factor (data not shown).

### Progesterone Increased Plasma Progestogens in PRKO >Wild-Type Male Mice

**Figure 2:**
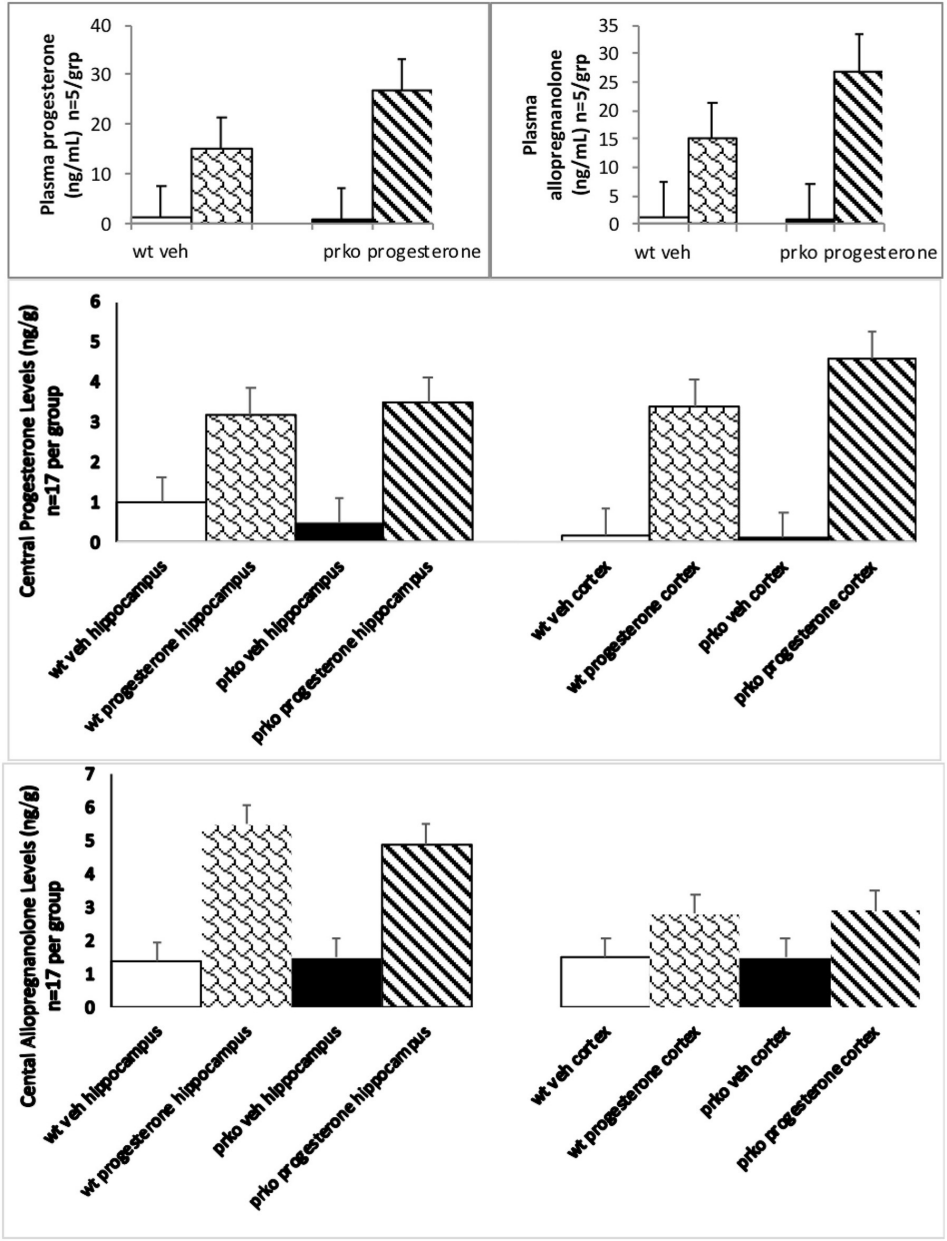
The results from the vehicle wildtype mice are represented by white bars, P_4_ wildtype by stippled bars, vehicle PRKO by black bars, and P_4_ PRKO mice are represented by diagonally striped bars. The **top left** panel represents plasma progesterone levels (*n* = 5/group) whereas the **top right** panel represents plasma allopregnanolone levels (*n* = 5/group). Progesterone administration significantly increased progesterone and allopregnanolone levels for PRKO > wild-type male mice. The **middle** panel represents central levels of progesterone in the hippocampus left side and cortex right side (*n* = 17/ this and all other brain groups). The **bottom** panel represents central levels of allopregnanolone in the hippocampus left side and cortex right side (*n* = 17/group). Progesterone administration significantly increased hippocampal and cortical progesterone and allopregnanolone levels for both wild-type and PRKO male mice.

Progesterone condition and genotype significantly interacted to influence circulating plasma progesterone [F_(1, 16)_ = 69.77, *P* < 0.0001] and allopregnanolone [F_(1, 16)_ = 28.59, *P* < 0.0001]. PRKO mice administered progesterone had significantly higher circulating progesterone (see Figure 2, top left) and allopregnanolone (see Figure 2, top right) levels compared to all other groups.

### Progesterone and Genotype Interacted With Progestogens in Cortex Not Hippocampus

Progesterone [F_(1, 64)_ =405.86, *P* < 0.01] and genotype [F_(1, 64)_ =5.27, *P* < 0.05] conditions significantly influenced hippocampal levels of progesterone. Progesterone administration produced hippocampal levels of progesterone around 3.25 ng/g compared to vehicle 0.58 ng/g. PRKOs had mean levels of progesterone around 2.1 ng/g compared to wild-types 1.8 ng/g (see Figure 2, middle right).

Progesterone condition and genotype significantly interacted to influence cortical progesterone levels [F_(1, 64)_ = 4.80, *P* < 0.03]. PRKO mice had significantly higher levels of progesterone in the cortex (see Figure 2, middle left) compared to all other groups.

### Progesterone Has Effects Irrespective of Genotype to Alter Central Allopregnanolone

Progesterone [F_(1, 64)_ = 202.10, *P* < 0.0001] condition significantly influenced hippocampal levels of allopregnanolone. Progesterone administration produced hippocampal levels of allopregnanolone around 5.2 ng/g compared to vehicle 1.4 ng/g (see Figure 2, bottom left).

Progesterone [F_(1, 64)_ =80.67, *P* < 0.0001] condition significantly influenced cortical levels of allopregnanolone. Progesterone administration produced cortical levels of allopregnanolone around 2.6 ng/g compared to vehicle 1.3 ng/g (see Figure 2, bottom right).

### Progesterone Increased Progestin Receptor Binding Sites in Hippocampus and Cortex Cortex of Wild-Type but Not PRKO Male Mice

**Figure 3:**
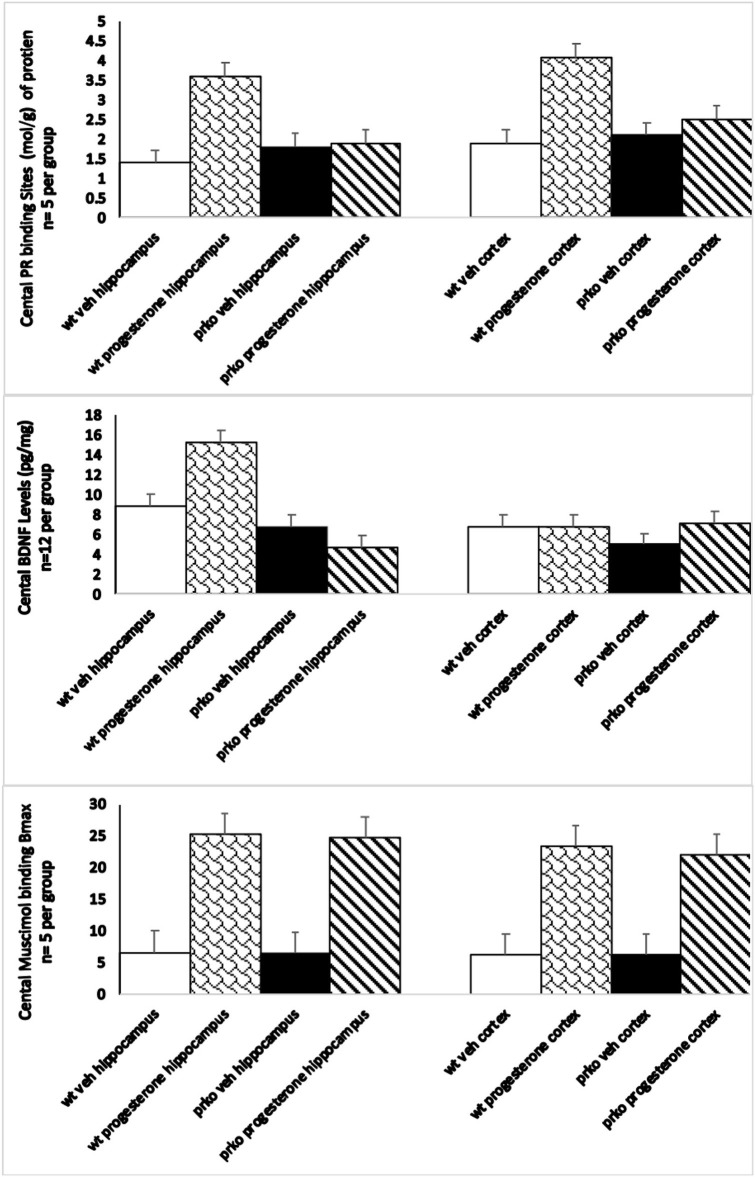
The vehicle wildtype mice results are represented by white bars, P_4_ wildtype by stippled bars, vehicle PRKO by black bars, and P_4_ PRKO mice are represented by diagonally striped bars. The **top** panel represents E_max_ progestin receptor binding (*n* = 5/group) in the hippocampus in the **left** panel, and cortex in the **right** panel. Only wild-type mice administered progesterone showed significant increases in progestin receptor binding. The **middle** panel represents BDNF levels (*n* = 12/group) in the hippocampus in the **left** panel, and cortex in the **right** panel. BDNF levels were only increased in progesterone administered wild-type mice in the hippocampus. The **bottom** panel represents maximum muscimol binding (*n* = 5/group) in the hippocampus in the **left** panel, and cortex in the **right** panel. Both wild-type and PRKO male mice administered progesterone showed significant increases in the maximal binding of the GABA_a_ agonist mucimol.

Progesterone condition significantly interacted with genotype to influence [^3^H] RU5020 Emax moles/g in the hippocampus [F_(1, 16)_ =12.14, *P* < 0.001]. Progesterone administration produced increased PR binding to ~3.5 moles/g compared to all other groups, which averaged 1.7 (see Figure 3, top left).

Progesterone condition significantly interacted with genotype to influence [^3^H] RU5020 Emax N/g in the cortex [F_(1, 16)_ =252.11, *P* < 0.0001]. Progesterone administration increased PR binding to ~4.3 moles/g among wild-types compared to all other groups, which averaged 2.2 (see Figure 3, top right).

### Progesterone Increased BDNF Levels in Hippocampus but Not Cortex of PRKO and Wild-Type Male Mice

There was a significant main effect of progesterone to increase BDNF levels in the hippocampus [F_(1, 46)_ =4.70, *P* < 0.008] irrespective of genotype (see Figure 3, middle left).

Progesterone, compared to vehicle administration, increased BDNF levels in the hippocampus. There was neither an effect of genotype, nor an interaction between genotype and hormone condition. These effects were not observed in the cortex (see Figure 3, middle right).

### Progesterone Increased E max Muscimol Binding in Cortex of PRKO and Wild-Type Male Mice

Progesterone administration significantly enhanced maximal ^3^H muscimol binding in the hippocampus irrespective of genotype [F_(1, 16)_ =2,030.41, *P* = 0.0001]. See Figure 3, left. There was no difference between muscimol binding of wild-type and PRKO mice.

Progesterone administration significantly enhanced maximal ^3^H muscimol binding in the cortex irrespective of genotype [F_(1, 16)_ =2,202.02, *P* = 0.0001]. See Figure 3, right. There was no difference between muscimol binding of wild-type and PRKO mice.

## Discussion

This study generally supports our a priori hypothesis that progesterone, compared to vehicle, to male PRKO and wild-type mice could improve cognitive performance. In the water maze task, wild-type mice tended to outperform PRKO mice, where wild-type mice had shorter latencies to find the hidden platform compared to PRKO mice. However, in the object recognition and T-maze tasks, progesterone improved performance in both the PRKO and wild-type mice. Progesterone administered to wild-type and PRKO mice higher progesterone levels in the hippocampus and cortex. On the contrary, PRKO mice had higher allopregnanolone in the hippocampus and cortex compared to wild-type mice. Moreover, wild-type, but not PRKO, mice had higher BDNF levels in the hippocampus with progesterone administration compared to vehicle administration. No differences were seen in the cortex for progesterone to increase BDNF levels. Yet, muscimol binding in cortex was similarly increased in wt and PRKO mice administered progesterone. Thus, progesterone's actions in wild-type and PRKO male mice to enhance cognitive performance may be associated with allopregnanolone and/or BDNF expression, in the hippocampus, or cortical increases in allopregnanolone and GABA_A_ activity.

In the present study, progesterone improved cognitive performance in the object recognition and T-maze task among wild-type and PRKO mice. This finding extends previous published data. Progesterone treatment immediately post-training enhances object recognition in young (14), middle-aged, and aged (64) mice. Also, progesterone administration to young and/or aged mice improved cognitive performance in these tasks (13, 60). However, in the water maze task, wild-type mice tended to outperform PRKO mice. There are beneficial effects of post-training progesterone on spatial memory consolidation in the Morris water maze in mice (64). In addition, it has been previously observed that aged wild-type mice outperform PRKO mice (57). Although, PRKO mice have reduced levels of PR binding (65, 66), there are significant increases in cognitive behavior of progesterone administered PRKO and wild-type mice concomitant with increased levels of allopregnanolone in the hippocampus (29). Progesterone can also have beneficial effects in other behaviors. For example, progesterone has beneficial effects for sexual behavior, motivation, anxiety, response to drugs of abuse (24) and also, depressive-like behaviors (54). Thus, progesterone exerts beneficial effects on cognition as well as other behaviors.

Progesterone can also have neuroprotective effects in the brain. The neuroprotective effects of progesterone have been demonstrated in rodent models of neurodegeneration (67), brain ischemia/stroke (68–71), and traumatic brain injury (TBI) (72–78). Moreover, progesterone limits the extent of tissue damage and the impairment of motor functions in an animal model of stroke (77). Furthermore, progesterone has neuroprotective and cognitive enhancing effects however progesterone also can have beneficial effects in other disorders (i.e., Alzheimer's, Parkinson etc.). Thus, progesterone can have neuroprotective and cognition enhancing, which support the purpose of such investigations.

BDNF levels were increased in the hippocampus with progesterone administration to wild-type, but not PRKO, mice compared to vehicle administration. As such, these findings add and extend the literature on interactions between progesterone and growth factors. In support, progesterone failed to elicit an increase in BDNF in PRKO mice but induced an increase in BDNF levels of wild-type mice (79). This evidence has suggested that classical intracellular/nuclear PR would be the principle mediator of the effects of progesterone on BDNF expression because this effect was inhibited by the pharmacological inhibitor of PRs, Mifepristone, and was lost in PRKO mice (79). In addition, levels of BDNF in the cortex and hippocampus were lowest among mice administered a synthetic progestin, medroxyprogesterone acetate, that does not act like progesterone to induce allopregnanolone synthesis, suggesting a downstream role of this growth factor (12, 79). Indeed, progesterone increased BDNF levels; however, this may only be possible by way of its metabolite, allopregnanolone. Furthermore, among young cycling rodents, manipulation of allopregnanolone synthesis in the midbrain alters expression of BDNF in the hippocampus (61). Others have noted that allopregnanolone alters BDNF expression in other limbic structures, such as the amygdala and hypothalamus, in addition to the hippocampus (80). Together, production of allopregnanolone in the hippocampus may be required for progesterone's mnemonic effects to increase BDNF levels.

In the present study, allopregnanolone levels were increased in the hippocampus and cortex of PRKO and wild-type mice. This may be due to increased activity of the stress axis, which can interact to alter circulating and brain levels of steroids (e.g., neurosteroids), particularly among mice. Though PRKO mice had increased levels of allopregnanolone, this was not associated with increased BDNF levels, which support the notion that PRs may not be involved for cognitive improvements with progesterone administration. It must be noted that levels of allopregnanolone and progesterone are very low in male mice, but they are not completely absent. This may be because there can be *de novo* synthesis of allopregnanolone in the brain itself, in addition to metabolism of circulating progesterone. Indeed, it may be that these levels of allopregnanolone were produced via actions in the hippocampus, cortex and/or other regions in this circuit involved in the behaviors observed. Another consideration is that the behavioral effects of PRKO mice may be less related to effects on production of allopregnanolone, or even other steroids that can be derived in the periphery or brain (progesterone), but may relate more to different rates of clearance of neuroactive steroids. Moreover, these effects may be related to increased rates of progesterone conversion to allopregnanolone, which is known to increase following environmental stressors (e.g., cold water swim, restraint) as well as social challenges (e.g., mating) and mitigates stress responding [reviewed in (24, 81, 82)]. Furthermore, this may explain the difference that was shown in the water maze task (latency to find the platform). The water maze requires a high degree of physical activity (swimming), remembering where a hidden escape platform is located in the pool, and a probe trial. Although this was not directly tested in the present study, the water maze task may be associated with an increased stress response in PRKO mice, which could lead to higher levels of allopregnanolone in circulation among both progesterone and vehicle administered mice. Thus, clearance of neurosteriods and/or stress responsiveness may play an important role in allopregnanolone production. Further investigation of this is substantiated.

## Conclusion

In conclusion, progesterone can have beneficial effects for cognitive performance among males. An important piece to this story may be progesterone's metabolite, allopregnanolone, and effects on BDNF levels in the hippocampus or GABA_A_ receptors in the cortex.

## Implications

Progesterone and its neuroactive metabolite, allopregnanolone, cognitive effects' among males is not well-understood and is addressed in this study. Progesterone (4 mg/kg, or oil vehicle SC) administered post-training in hippocampus and/or cortex tasks to wild type or mice lacking functional nuclear PRs. In the water maze task, wild-type mice tended to outperform PRKO mice. Progesterone enhanced performance of irrespective of genotype in the water maze, object learning and T-maze. Progesterone, but not vehicle, increased progesterone and allopregnanolone levels of wild-type and PRKO mice; albeit, PRKO mice had higher allopregnanolone levels than did wild-type in the hippocampus and cortex. Wild-type mice administered progesterone, but not vehicle, had increased BDNF levels in the hippocampus compared to PRKOs. Thus, male mice can be responsive to progesterone for learning/memory, and such effects do not require PRs, but may be associated with allopregnanolone and BDNF levels in the hippocampus or GABA_A_ activity in the cortex.

## Data Availability Statement

The raw data supporting the conclusions of this article will be made available by the authors, without undue reservation.

## Ethics Statement

The animal study was reviewed and approved by Institutional Animal Care and Use Committee.

## Author Contributions

AW: data production. VL: formal analysis. CF: funding acquisition, supervision, concept, and original draft. All authors contributed to the article and approved the submitted version.

## Conflict of Interest

The authors declare that the research was conducted in the absence of any commercial or financial relationships that could be construed as a potential conflict of interest.
